# Supplementary material for: Conserved Yet Divergent Smc5/6 Complex Degradation by Mammalian Hepatitis B Virus X Proteins
Source: Int J Mol Sci. 2025 Jul 15;26(14):6786. doi: 10.3390/ijms26146786 (PMC12296160; doi:10.3390/ijms26146786)
Supplement: Supplementary file 1 [file ijms-26-06786-s001.zip › 250714 Supplementary Table Ver proof AS2.pdf]

Table S1. Genetic distance of mammalian Smc6 based on amino acid sequence

The number of amino acid substitutions per site from between sequences are shown.

No		1	2	3	4	5	6	7	8	9	10	11	12	13	14	15	16	17	18	19	20	21	22	23	24	25	26
1	NP_001135758.1_Homo_sapiens																										
2	AWK27963.1_Homo_sapiens_293T	0.00																									
3	AWK27960.1_Homo_sapiens_HepG2	0.00	0.00																								
4	XP_054404336.1_Pongo_abelii	0.01	0.01	0.01																							
5	XP_055234727.1_Gorilla_gorilla_gorilla	0.00	0.01	0.00	0.01																						
6	AWK27961.1_Chlorocebus_tantalus_Cos-7	0.02	0.02	0.02	0.02	0.02																					
7	AWK27957.1_Chlorocebus_pygerythrus_Vero	0.02	0.02	0.02	0.02	0.02	0.00																				
8	XP_007969584.1_Chlorocebus_sabaeus	0.02	0.02	0.02	0.02	0.02	0.00	0.00																			
9	XP_005576553.2_Macaca_fascicularis	0.02	0.02	0.02	0.02	0.02	0.00	0.00	0.00																		
10	XP_014967240.1_Macaca_mulatta	0.02	0.02	0.02	0.02	0.02	0.00	0.00	0.00	0.00																	
11	AWK27958.1_Aotus_trivirgatus_OMK	0.04	0.04	0.04	0.04	0.04	0.04	0.04	0.04	0.04	0.04																
12	AWK27962.1_Saguinus_oedipus_B95a	0.04	0.04	0.04	0.04	0.04	0.04	0.04	0.04	0.04	0.04	0.02															
13	AWK27959.1_Mustela_putorius_MPF	0.06	0.06	0.06	0.06	0.06	0.06	0.06	0.06	0.06	0.06	0.08	0.08														
14	XP_023107850.1_Felis_catus_Isoform_X1	0.08	0.07	0.08	0.08	0.08	0.08	0.08	0.08	0.07	0.07	0.08	0.08	0.04													
15	XP_003984536.2_Felis_catus_isoform_X2	0.08	0.07	0.08	0.08	0.08	0.08	0.08	0.08	0.07	0.07	0.08	0.08	0.04	0.00												
16	XP_038416754.1_Canis_lupus_familiaris	0.07	0.07	0.07	0.07	0.07	0.07	0.07	0.07	0.07	0.07	0.07	0.08	0.03	0.01	0.01											
17	XP_027629216.1_Tupaia_chinensis	0.07	0.07	0.07	0.07	0.07	0.07	0.07	0.07	0.07	0.07	0.07	0.07	0.08	0.07	0.07	0.06										
18	XP_038968116.1_Rattus_norvegicus	0.09	0.09	0.09	0.09	0.10	0.10	0.10	0.10	0.09	0.10	0.10	0.10	0.10	0.09	0.09	0.08	0.09									
19	NP_079971.2_Mus_musculus	0.10	0.10	0.10	0.10	0.10	0.10	0.10	0.10	0.10	0.10	0.10	0.10	0.11	0.09	0.09	0.09	0.09	0.02								
20	XP_002691521.2_Bos_taurus	0.08	0.08	0.08	0.08	0.09	0.09	0.09	0.09	0.08	0.08	0.09	0.09	0.08	0.07	0.07	0.06	0.07	0.10	0.10							
21	XP_003125390.1_Sus_scrofa	0.08	0.09	0.08	0.08	0.09	0.08	0.08	0.08	0.08	0.08	0.08	0.09	0.08	0.07	0.07	0.06	0.07	0.10	0.11	0.06						
22	XP_032980470.1_Rhinolophus_ferrumequinum	0.09	0.09	0.09	0.09	0.09	0.09	0.09	0.09	0.09	0.09	0.09	0.09	0.09	0.08	0.08	0.07	0.08	0.10	0.11	0.08	0.08					
23	XP_016009374.1_Rousettus_aegyptiacus	0.09	0.09	0.09	0.09	0.10	0.09	0.09	0.09	0.08	0.09	0.09	0.09	0.09	0.08	0.08	0.08	0.08	0.11	0.11	0.09	0.08	0.06				
24	XP_006910309.1_Pteropus_alecto	0.09	0.09	0.09	0.09	0.09	0.09	0.09	0.09	0.08	0.08	0.09	0.09	0.08	0.07	0.07	0.07	0.07	0.10	0.10	0.08	0.08	0.06	0.03			
25	XP_023384272.1_Pteropus_vampyrus	0.10	0.10	0.10	0.10	0.10	0.10	0.10	0.10	0.09	0.10	0.10	0.10	0.09	0.08	0.08	0.08	0.09	0.11	0.12	0.09	0.09	0.08	0.04	0.01		
26	XP_028932004.1_Ornithorhynchus_anatinus	0.39	0.39	0.39	0.39	0.40	0.39	0.39	0.39	0.39	0.39	0.40	0.40	0.39	0.39	0.39	0.39	0.39	0.41	0.41	0.40	0.40	0.39	0.40	0.41	0.43	

Table S2. Synthesized DNAs for generating plasmids encoding X protein

**X protein**	**Codon-optimized DNA sequence**
HBV(genotype A)<br>Accession# LC488828.<br>Host: human	ATGTACCCTTACGATGTACCTGACTACGCGACCGGTGCCGCT<br>CGACTTTATTGCCAACTTGATCCATCCCGCGACGTGTTGTGT<br>CTCAGACCCGTTGGCGCCGAATCTCGAGGTCGCCCACTTTCA<br>GGACCCCTTGGTACATTGAGTTCTCCATCTCCGAGCGCTGTA<br>CCGGCGGACCACGGGGCTCATTTGAGTTTGAGGGGTCTCCC<br>AGTGTGTGCTTTCAGCAGTGCCGGCCCGTGCGCGCTTCGATT<br>CACTTCAGCAAGATGTATGGCTACGACTGTCAATGCTCATCA<br>AATCCTCCCAAAGGTCCTCCACAAAAGAACGCTTGGGCTTCC<br>GGCCATGTCTACTACTGATCTTGAGGCGTACTTCAAGGACTG<br>CGTCTTTAAGGATTGGGAGGAATTGGGGGAGGAAATTCGCC<br>TTAAAGTATTCGTGCTGGGGGGTTGCAGACACAAACTTGTAT<br>GCGCTCCTGCCCCGTGCAACTTCTTCACTTCCGCATAA
HBV(genotype D)<br>Accession# YP_009173867.1<br>Host: human	ATGTACCCTTACGATGTACCTGACTACGCGACCGGTGCCGCA<br>CGCCTCTGTTGCCAACTTGATCCAGCACGGGATGTTCTGTGC<br>CTTCGGCCAGTCGGCGCTGAAAGTTGTGGACGGCCGTTCTCC<br>GGCTCCCTCGGAACGCTCTCCTCCCCCTCCCCCTCAGCCGTC<br>CCTACAGATCATGGAGCTCACCTTTCTCTTCGCGGGCTTCCG<br>GTATGCGCGTTTTCTTCTGCTGGACCTTGCGCGTTGCGCTTTA<br>CATCTGCTAGGAGAATGGAAACAACGGTTAACGCGCACCAG<br>ATACTCCCGAAGGTTTTGCACAAAAGGACCCTGGGTCTTAGT<br>GCCATGAGCACTACTGATCTCGAGGCATACTTTAAGGACTGC<br>TTGTTCAAGGATTGGGAGGAACTTGGGGAAGAGATCAGATT<br>GAAGGTGTTCGTCTTGGGGGGATGTCGGCATAAGCTCGTGT<br>GTGCACCTGCACCCTGCAATTTCTTCACGTCTGCTTAG
HBV(genotype G)<br>Accession# BAD91282.1<br>Host: human	ATGTACCCTTACGATGTACCTGACTACGCGACCGGTGCAGCC<br>CGGCTCTGTTGTCAGTTGGATCCCAGCCGAGACGTTCTCTGC<br>TTGCGGCCAGTTAGCGCAGAGTCATCTGGACGCCCGCTGCCC<br>GGACCTTTTGGTGCACTCTCTCCTCCAAGTCCCTCTGCAGTC<br>CCCGCAGATCATGGCGCTCACCTGTCATTGCGCGGTTTGCCA<br>GTATGCGCCTTCTCCTCAGCAGGTCCGTGCGCGCTTCGATTC<br>ACGTCAGCACGGTACATGGAAACGGCAATGAATACGAGCCA<br>CCATCTCCCCCGACAACTTTACAAACGCACCCTCGGTCTTTT<br>CGTTATGAGTACCACTGGTGTCGAGAAGTACTTTAAAGACTG<br>TGTCTTCGCTGAATGGGAGGAGTTGGGCAACGAGAGCCGAC<br>TGATGACTTTCGTGCTGGGCGGATGCAGACATAAGCTCGTGT<br>GTGCTCCGGCCCCCTGTAATTTCTTTACTTCAGCTTAA
HBV(genotype H)<br>Accession# BAJ15483.1<br>Host: human	ATGTACCCTTACGATGTACCTGACTACGCGACCGGTGCCGCT<br>AGGCTGTGCTGCCAACTTGATCCCGCCCGCGACGTGCTGTGT<br>TTGCGGCCAGTAGGTGCTGAAAGCTGCGGTCGGCCACTTTCC<br>TGGTCCCTCGGGGCTTTGCCCCCGTCATCACCTCCAACAGTT<br>CCTGCTGACGACGGATCTCACTTGAGTCTTCGGGGGTTGCCC<br>GCGTGCGCGTTCAGTTCCGCTGGTCCTTGTGCGTTGCGATTT<br>ACGAGTGCTAGGAGAATGGAAACGACTGTCAACGCTCCCTG<br>GAACTTGCCCACTACGCTGCACAAACGAACATTGGGTCTGTC<br>TCCCCGCTCCACGACTTGGATAGAAGAGTACATTAAAGACT<br>GTGTTTTCAAAGACTGGGAGGAAAGTGGGGAGGAACTTCGC<br>CTGAAGGTGTTTGTGCTTGGCGGTTGTAGACACAAACTCGTG<br>TGTTCCCCGGCCCCTTGTAACTTCTTCACTAGCGCATAG

DCHBV(KT116)<br>Accession# LC668427.1<br>Host: Domestic cat	ATGTACCCTTACGATGTACCTGACTACGCGACCGGTGCAGCA<br>CGGCTGCGCTGCGAACTCGATCCTTCTGGTCGGGTCCTGCGG<br>TTGAGACCATTCATTAGTGAATCCAGCGGACGCGCGGTTAG<br>CCGAACTGCACGCTTGCCAGACCTGAGCCCCTCAGTTGCGGT<br>TTCAGCGACACTGCGGGCCAGGGAATCTCTGAGGGGTATAC<br>CTGCCTGTCTCACGTCACCAGAGGGCCCTTGTGTTTTGAGAT<br>TTACCTGCGCTGATAGTAGACGGTGCATGGAAGCAGCAATG<br>ATTGGCTTGGTCCCAGCACTGCTTGCTCGCCAACTTGGCTTC<br>GGGACTTGGCAGCCGGATGTATGGACGCTTCGGCTTCGCGA<br>TCTTTTGTTGGTCGAGTGGGAGGAAGAAGGACTGACGCCGC<br>GGTTGTGTACTTATCTTGTAACGGGGTGCGCTCATAAAACGC<br>TTCACACTCGATAG
Domestic donkey HBV<br>Accession# QMV34684.1<br>Host: Domestic donkey	ATGTACCCTTACGATGTACCTGACTACGCGACCGGTGCGGCT<br>CGCCTGAGGTGTCAACTGGACCCAACTGGCCGGGTACTCCA<br>CCTCCGACCCTTTACTTCCGAATCTTGTAGAAGAACTTTGGC<br>AGGTACTGCCGGGGCACCAGATCTCCCAGCAGCGGACCTCC<br>TTCAAGCGGATCACCGGACTCATCTTAGGGTTCGACGCTTGC<br>CTGCTTGCTGTTTCTCTTCTCGCGGTCCGTGTGTGCTTAGGTT<br>CACATGCGCGGACCTTAGCCGACGAATGGAAGCCCCGATGA<br>ACCTCGTTCAATATCTGGGGAAAAGGGCGCGGGGTCTTCAG<br>CATCCGCCCGGTGATTCCTATTGCCAACATGAACTTTGGACA<br>CAATGGGAGGAGAATGGTTGGTCAGACAGAATCTATACTTA<br>CGTGTTGGGAGGATGCAGACACAAATGGCTTTACCCACTTTA<br>G
Asian grey shrew HBV<br>Accession# YP_010796421.1<br>Host: Asian grey shrew	ATGTACCCTTACGATGTACCTGACTACGCGACCGGTGCTGCA<br>AGAATGCTCTTCGATCTCGACCCTGCTACAGGAGCTGTACGC<br>CTTCGCCCATTTCTCACTGAACCCCGCGGACGAGGGGAACA<br>GACACCGCGCCCGACTTCCTCTCCGACAACGTCAGCCCTGTC<br>TTCTTTCCTTGGAAGTCGGTCTTCCTGGCGGCGCTTGCCAAG<br>CTGCGCCGACTCTCCATTCGGCCCATGTACTTTGCGGTTCAC<br>GTTCGCAGAGCTGGGAAACTTGCAGACACCAATGAACTCAG<br>TGACCTTCATCAGTTGTCGGTCAAGGGGAGCCCATCTGAAGT<br>GCCGGAGGCAACAGAAGAATTGGACCTGGTATTTCTGGACA<br>CATCATAATGCGAACAACACGCACCATTTGTGGCTTATGTGC<br>TACGGAGGTTGTAGGCATAAATAG
Capuchin monkey HBV<br>Accession# YP_009666527.1<br>Host: Capuchin monkey	ATGTACCCTTACGATGTACCTGACTACGCGACCGGTGCAGCC<br>AGACTTTGTTGCCAACTGGACCCTGCCAGGGATGTTCTTTGT<br>CTCCGACCTGTAACTGCCGAGCCATGTGGACGACCCTTCAGC<br>GGTTCTGCTCGGACATCCGCTCCGGCAGCTGCGGCAGCCCTG<br>CCCTCTATTGATGGAGCATATCTGTCCCTTCGAGGGCTTCCT<br>AGTTGCGCTTTCTCATCCTCAGGGCCCTGCGCCTTGAGGTTT<br>ACAAGTGCGCGACGAATGGCTACACCGATGAATAGTAGAGA<br>TCTGGTCCAACAACTCTATAATCGGACGTTGGGTCTTGCTCC<br>TCTCTCCACTGGGCAGTGGGAACGGCACTTTAAAGATCTTTT<br>GTTCGAGGAATGGGAGGAACTCGGTGTTGAGTTCAGGTTGA<br>AAGTATTCGTGCTGGGGGGTTGTCGCCATAAGCTCGTTTGCA<br>GTGTGCAACCTTGCATATTCTTCACTAGTGCCTAA
Orangutan HBV<br>Accession# AAF33122.1<br>Host: Orangutan	ATGTACCCTTACGATGTACCTGACTACGCGACCGGTGCTGCC<br>CGCCTTTGTTGTCAGTTGGACCCGGCTCGAGATGTCCTTTGC<br>CTTCGGCCAGTGGGAGCTGAGAGTAGAGGAAGGCCGTTCCC<br>AGGCAGTATTGGTGCTCTGCCCCCACCATCTCTGAGCGCGGT<br>ACCGGCCGACCACGGAGCCCACCTTAGCCTTCGGGGTTTGC<br>CGGTATGCGCTTTTTCTTCAGCGGGGCCTTGTGCGTTGAGGT

	TCACGAGCGCCAGGTGCATGGAGACCACCGTAAATGCGCCT<br>AGAAATCTCCCTAAGGTCCTGCATAAGAGAACATTGGGCCT<br>TTCCACTATGTCTACTACGCGAATCGAAACGTACTTTAAGGA<br>TTGTGTGTTTAAGGATTGGGAAGAACTTGGAGAGGAGATCC<br>GGTTGAAGGTTTTTGTCTTGGGTGGATGTAGGCATAAATTGG<br>TGTGTTCTCCCGCGCCTTGCAACTTTTTTACAAGTGCA<u>TGA</u>

Table S3. Primers used for generating a plasmid encoding Smc6 protein

Host	Direction	Sequence (5′-3′)
Human	Forward	TGACTACGCGACCGGTGCCAAAAGAAAGGAAGAAAATTTT
	Reverse	AAAAAGATCTGCTAGCTCACCTTTGGTCATCATCTTCTTCT
Feline	Forward	GATGTACCTGACTACGCGACCGGTGCCAAAAGAAAGGAAGAAAA
	Reverse	AAAAAGATCTGCTAGCTCAGCTCCGGTCTTCTTCCTCC
Feline (+KVRNT)	Forward	AAAGTAAGGAACACCAAATTCTTTATGAAAGCAAC
	Reverse	GGTGTTCCTTACTTTGTATTTGTCTCCCTCATTTT
