# Supplementary figures and images for: Conserved Yet Divergent Smc5/6 Complex Degradation by Mammalian Hepatitis B Virus X Proteins
Source: Int J Mol Sci. 2025 Jul 15;26(14):6786. doi: 10.3390/ijms26146786 (PMC12296160; doi:10.3390/ijms26146786)

Figure S1

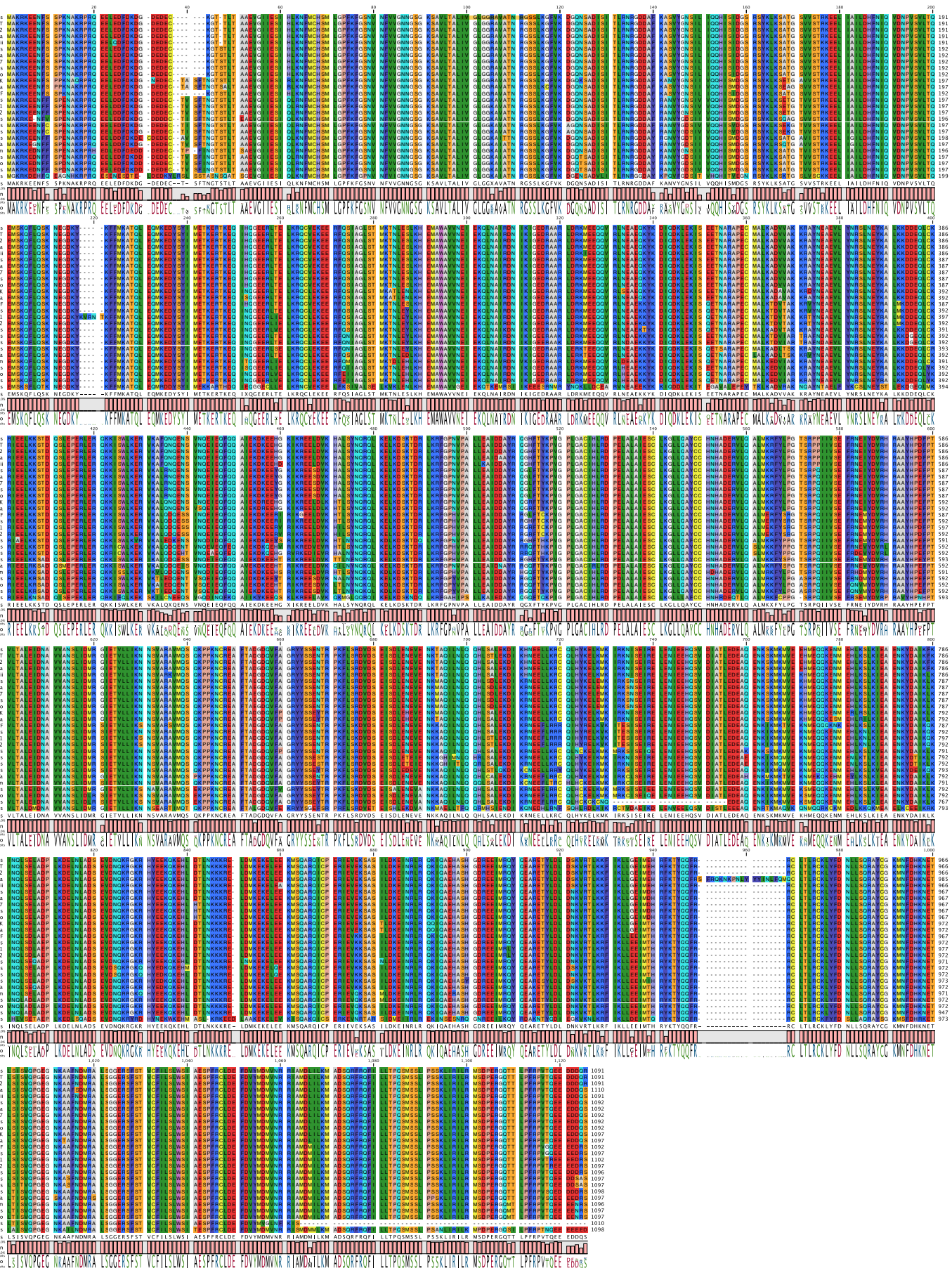

**Figure S2**

Supplement: Supplementary file 1 [file ijms-26-06786-s001.zip › 250714 Smc6 paper Supplementary Figures proof AS3.pdf]
